# 4-week stretching program after submaximal strength exercise affects performance but not heart rate variability and lactate clearance. An exploratory study

**DOI:** 10.3389/fspor.2024.1424756

**Published:** 2024-06-18

**Authors:** M. Alessandria, S. Angilletta, I. Pivetta, B. Annone, S. Cravanzola, A. De Giorgio

**Affiliations:** ^1^ Department of Life Sciences and Systems Biology, University of Turin, Turin, Italy; ^2^ A.S.D. “SportTiVà?”, Turin, Italy; ^3^ Faculty of Psychology, eCampus University, Novedrate, Italy

**Keywords:** HRV, HRR, biological marker, physiological marker, back squat repetitions

## Abstract

Previous research has demonstrated that stretching can enhance athletic performance and induce cardiovascular adaptations. This study aims to assess whether a 4-week preventative stretching routine can enhance heart rate variability and heart rate recovery, faster blood lactate clearance, and improve performance following submaximal strength exercises. Twenty-four healthy adults were recruited and randomly allocated to either the experimental group or the control group. Both groups engaged in submaximal strength exercises (5 sets to voluntary failure at 60% of 1RM) comprising bench press and back squat exercises under baseline conditions and after stretching protocol. The experimental group followed the Stretching Protocol, while the control group adhered to their regular training routine. ANOVA analysis revealed a significant pre-post interaction effect between groups in the variable of squat repetitions, although no notable pre- or post-differences were observed in heart rate variability, heart rate recovery, blood lactate concentration, or bench repetitions in either group. A 4-week preventative stretching program does not appear adequate to enhance lactate clearance and cardiovascular adaptation after submaximal strength exercises in resistance-trained individuals compared to the control group. However, it is plausible that such a stretching routine may mitigate muscle fatigue, though further investigation is warranted to substantiate this hypothesis.

## Introduction

Engaging in a single session of high-intensity resistance training can lead to immediate fatigue and a temporary decline in performance (1–3). On the other hand, brief periods of consistently elevated training beyond the usual level followed by adequate recovery can lead to a rebound effect called functional overreaching (4). Conversely, prolonged exposure to excessive training, without adequate recovery, can result in a diminished adaptive response and long-term decline in performance, referred to as non-functional overreaching (5). Functional overreaching and non-functional overreaching are respectively defined in literature as “A short-term decrease in performance lasting days to weeks with subsequent performance supercompensation after a period of recovery” and “Performance decrement is observed over weeks to months, and while full recovery is achieved (although not always), no super-compensation effects are achieved” (6–9). It is important to highlight that the second condition could lead to both overtraining syndrome (9)—defined as “Long-term reductions in performance capacity observed over several months”—and injuries (10, 11). These conditions all share an inadequate recovery between the exercises performed. In both physical activity and sports, stretching is among the practices used for proper recovery post-exercise (12). Several authors have highlighted its significant effects on blood lactate clearance if carried out during the recovery phase (13–15). Substantial microcirculatory events have been observed up to 10 min after stretching itself, such as post-stretch hyperaemia (16). Subsequent evidence has shown that these microcirculatory events can also be observed in the long term: after 4 weeks of muscle stretching, an increase in blood flow in the stretched limb was observed during exercise on the treadmill, compared to the contralateral non-stretched limb (17). This evidence demonstrates that stretching exercises administered after a training session can influence the recovery capacity, even regarding peripheral muscle microcirculation (18, 19) because of the production and release of nitric oxide (20). Stretching has also been shown to have an impact on the cardiovascular system's health. Indeed, this type of training has been demonstrated to lead to a beneficial alteration in the autonomic system. Mueck-Weymann and colleagues (21) have highlighted, after a 28-day training period with a standardized 15-min stretching program, that healthy male athletes experienced a significant decrease in heart rate, a significant decrease in the Low Frequency/High Frequency ratio (indicating a shift towards vagal dominance), and a significant increase in indicators related to the improvement of heart rate variability (HRV). Farinatti and collaborators (22) found that multiple-set flexibility training sessions improve HRV and autonomic balance in the post-exercise recovery period for individuals with low flexibility levels, demonstrating that stretching exercises can positively impact autonomic activity, potentially offering cardiovascular protection. To the best of our knowledge, it is not possible to establish whether these results can also be obtained with a preventive stretching program capable of stimulating adaptation to allow faster recovery during physical activities that require phases of intense effort alternating with phases of rest. Furthermore, regarding performance, the positive effects of stretching in programs lasting more than 6 weeks are evident (23), but it has not yet been demonstrated whether these effects would also be observed in a program of shorter duration. For all these reasons, we aimed to investigate how a 4-week stretching program, administered to a sample of trained individuals, compared to a non-stretching control group, can influence: (i) performance (i.e., number of bench press and squat repetitions); (ii) indices of cardiovascular system function, such as HRV and heart rate recovery (HRR); (iii) promote a more rapid clearance of blood lactate.

## Materials and methods

### Participants

Twenty-four healthy adult resistance-trained men and women were recruited after a verbal invitation presented to patrons of a fitness centre (demographic characteristics in Table 1).

**Table 1:** Demographic characteristics of the groups involved in the study.

Group	Gender	Age (years)	Height (m)	BMI	Maximum load bench (kg)	Maximum load squat (kg)
		Mean (±SD)	Mean (±SD)	Mean (±SD)	Mean (±SD)	Mean (±SD)
Experimental	6M; 6F	29.75 (±5.41)	1.72 (±0.08)	22.60 (±2.41)	67.79 (±27.33)	100.67 (±40.74)
Control	5M; 5F	27.40 (±5.89)	1.70 (±0.12)	22.62 (±3.65)	68.50 (±34.81)	94.10 (±34.55)

BMI, body mass index; i.e., the measure of body fat based on height and weight that applies to adult men and women.

In the absence of evidence regarding the effects of preemptive stretching on lactate clearance, the sample size was calculated based on previous papers that administered a stretching protocol during the recovery phase that set the effect size at 1.20 (13). It was used *t*-test for the independent sample, with an α level of 0.05 and a power of 0.80. The sample size was determined using the G*Power 3.1.9.6 version. The recruitment phase involved an interview with the researcher responsible for the study with each volunteer to identify the exclusion and inclusion criteria. The exclusion criteria were the following: use of anabolic steroids or any other type of doping drug, previous injuries that occurred in the short period preceding the trial (dysmorphisms of the lower and upper limbs, recent fractures of the skeleton, and recent muscle injuries to the lower and upper limbs, recent skeletal fractures and recent muscle injuries of the lower and upper limbs), adverse medical conditions (coagulation, liver and diabetic diseases, vitamin D deficiency, anticoagulant therapies). The inclusion criteria were the ability to perform back squats and bench presses following the International Powerlifting Federation (IPF) guidelines (24), at least 3 years of experience in resistance training programs, and practicing back squats and bench press at least once a week to achieve maximum metabolic exhaustion. Other inclusion criteria were 1RM (one repetition maximum) back squats at 1.5 times body mass for men and 1.0 times body mass for women in the past month (25) and 1RM bench presses at 0.9 times body mass for men and 0.5 times body mass for women in the past month (26). To calculate 1RM Seo et al. (27) procedure was adopted since they showed a high reliability regardless of muscle group location or gender (27). Everyone was asked to maintain their eating and exercise habits during the trial period. All participants gave their informed consent to the experimental procedures, following the Declaration of Helsinki and approved by the Institutional Ethics Committee of the University of Turin, protocol number 0242983.

### Equipment

Blood lactate concentration was measured with a portable lactate analyzer (Accutrend Plus®, Roche Diagnostics Gmbh, Mannheim, Germany; (28), while HRV and HRR were measured with a chest strap device [Polar H10, Polar Electro Oy, Kempele, Finland; (29)]. To process the HRV data, two software were used: the Elite HRV app (version 5.5.1) and the Kubios HRV standard [version 3.5.0; (30, 31)]. All participants used an IPF-approved belt (10 cm wide and 10 mm thick), IPF-approved knee pads, and, if necessary, IPF-approved wrist wraps. The shoes were footwear specially designed for powerlifting.

### Study design

All trials were performed in a fitness centre. Participants were randomly divided into two groups: an Experimental Group (EG) and a Control Group (CG). Randomization was performed by assigning a numerical code to each participant subsequently inserted into an Excel spreadsheet, where the Excel function = INDEX [array; RANDBETWEEN(1;24)] was used, which applies the Mersenne Twister algorithm (32). Before testing, participants had to stop training for at least 3 days and not have performed a heavy squat or bench press session in the previous week, so as not to experience delayed onset muscle soreness or drug-induced neurological fatigue induced by a heavy leg training session (33). The sequence of trials included measurements in the Baseline Condition (BC) and the After Stretching Protocol (ASP). Only the EG performed the stretching protocol, while the CG continued its regular training program. HRV was recorded at rest, before warming up. Afterward, participants were able to perform a self-selected warm-up session. At the end of the warm-up, participants performed 5 sets of bench press and back squat exercises to volitional failure at 60% of 1RM consecutively (34). Regarding to squat, we adhered to the rules of the IPF whereas for the bench press—allow participants to achieve a higher number of repetitions and consequently produce more lactate—we opted for the touch-and-go technique to. This approach is supported by a previous study on lactate response to varying power clean volume patterns (35). An operator counted the repetitions of the bench press and back squat in each set. According to the literature (36) we used a 2 min interval between sets of repetitions. To record an accurate measurement of HRR immediately after the end of the trials, a chest strap device was worn immediately before the start of the last set. HRR recording was performed for 1 min (37). During the study period, two CG participants decided to leave the study before the end and their data was deleted as they requested the deletion of any data connected to them.

### Stretching protocol

**Figure 1:**
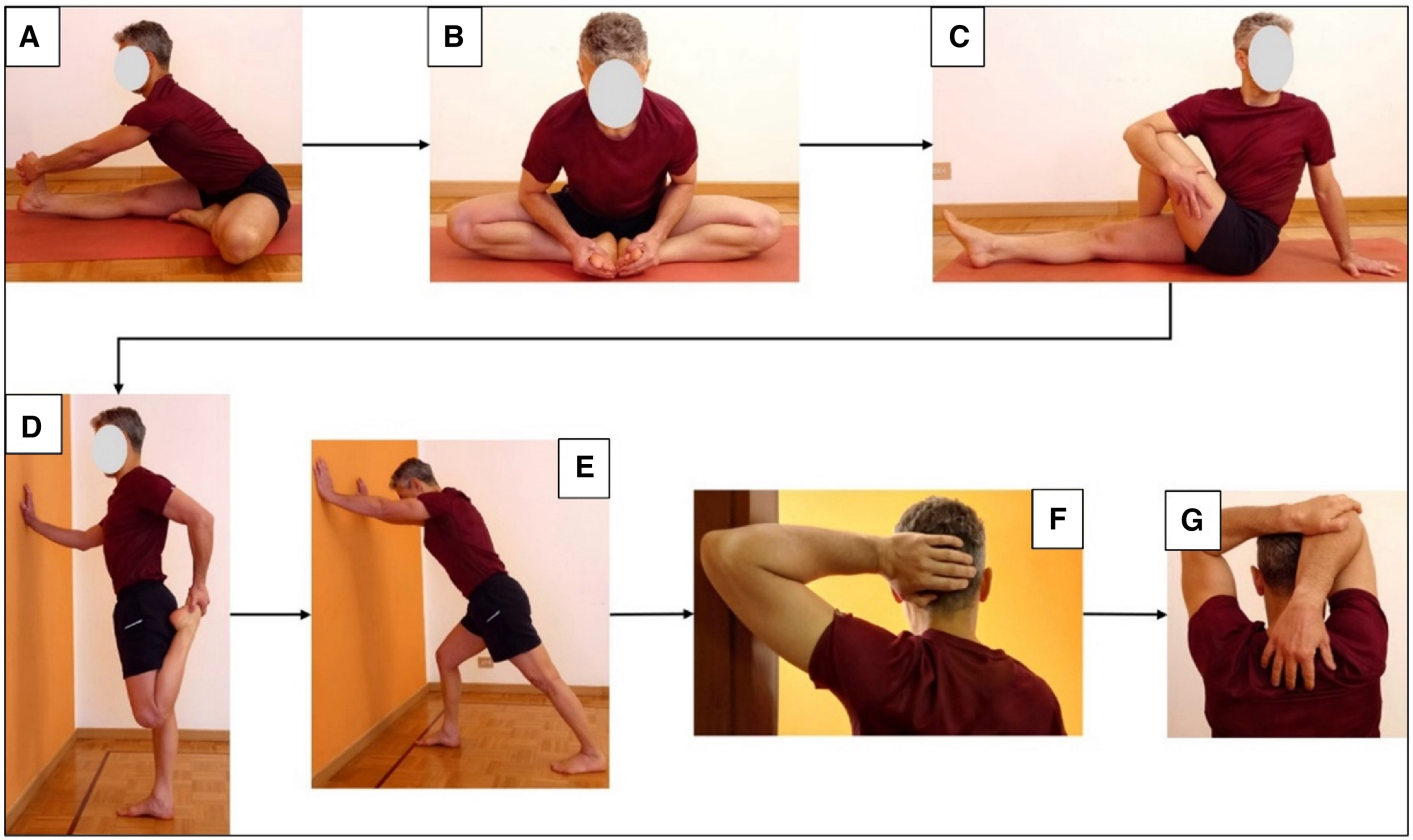
Stretching intervention sequence. (**A**) Harmstring (semibranosus, semitendinosus, and biceps femoris); (**B**) hip adductors (lungus, brevis, magnus); (**C**) gluteus (minimus, medius, maximus); (**D**) quadriceps; (**E**) sural triceps (gastrocnemius and soleus); (**F**) pectoralis (major, minor); (**G**) brachial triceps.

The EG participated in 20-min stretching sessions over four weeks. These sessions were conducted without prior warm-up to maximize the effects of muscle stretching (38). Each participant performed the sessions independently, ensuring they took place at the same time and location each day to maintain consistent environmental conditions. Each exercise lasted 45 s, totalling seven exercises performed three times a week. These exercises targeted the key muscles involved in a squat and bench press movements. At the end of the first circuit, the session was repeated a second time. According to Marchetti and colleagues (39), the intensity of the stretching session was set at 100% of a scale to evaluate the point of discomfort, where 0 = no stretching discomfort and 100% = maximum stretching discomfort imaginable. The stretching protocol included knee flexors performed in a sitting position with one leg straight and a straight back. The other leg was bent so that the soles of the feet rested against the middle of the thigh. In this position the participants had the trunk bent towards the extended lower limb while keeping the knee and back straight; hip adductor: in a sitting position the soles of the feet were brought together, with a straight back bent forward and keeping the knees apart; gluteus: in a sitting position with one leg straight, crossing the other leg over the straight leg with the sole resting on the ground. Keeping the back straight and one arm resting on the ground, the subject turned his trunk towards the bent leg, helping himself with the other arm to keep the leg and trunk together; quadriceps: in a standing position on one leg the participants held on something solid, the other knee was bent, and the heel was brought towards the buttock. The thighs were parallel to each other, and the abdominal muscles contracted; calf: in the upright position the participants supported themselves against the wall with their hands, one leg was brought backward making sure that the toes were pointing forward, the heel on the ground, and the knee straight. With a straight back, the pelvis was brought forward, keeping the heel on the ground and the knee tense; pectorals: in an upright position the participants were positioned with one hand behind the head forming an angle of approximately 45° behind the arm and forearm. The internal part of the elbow was placed on a fixed support, unbalancing the trunk anteriorly and taking a step forward with the opposite leg (40); triceps: in a sitting or standing position, the participants flexed their arm and forearm and moved their hand towards the shoulder blade. The elbow was grabbed with the opposite hand and pulled behind the head to increase arm flexion (41) (Figure 1).

### Statistical analysis

After performing the Shapiro-Wilk normality test, a parametric statistical analysis was used. The dependent variables considered were blood lactate concentration, number of repetitions of bench presses and squats (i.e., performance), HRR, and two summary measures of HRV: parasympathetic nervous system and sympathetic nervous system. The values used to analyze the HRR were the β coefficient of the linear regression model of the heart rate measurements in the first minute after the end of the trials (42). Partial eta square (*η*^2^) was interpreted accordingly: 0.01 small; 0.06 medium; and 0.14 large (43). The differences between the number of repetitions of bench presses and back squats, HRR, and HRV were assessed with mixed-model analysis of variance (ANOVA) with “Time” as within factor (two levels: Baseline and After) and “Groups” as between factor (two levels: Control and Experimental). Tukey's post-hoc among Groups was used. A linear mixed model for repeated measures was used for the blood lactate concentration. More specifically, we applied an unstructured variance-covariance matrix. To account for the correlation of measurements on the same subject, the Bonferroni correction for multiple comparisons was applied and α was set at 0.05. IBM SPSS Statistic 28.0 was used to analyze the data.

## Results

### Number of bench press repetitions

No significant pre-post differences were observed for this outcome between BC and ASP (main effect: *F*_1,20 _= 1.479, *p* = 0.238; interaction Test*Groups: *F*_1,20 _= 0.764, *p* = 0.392), as well as between groups (*F*_1,20 _= 1.003, *p* = .329).

### Number of squat repetitions

**Figure 2:**
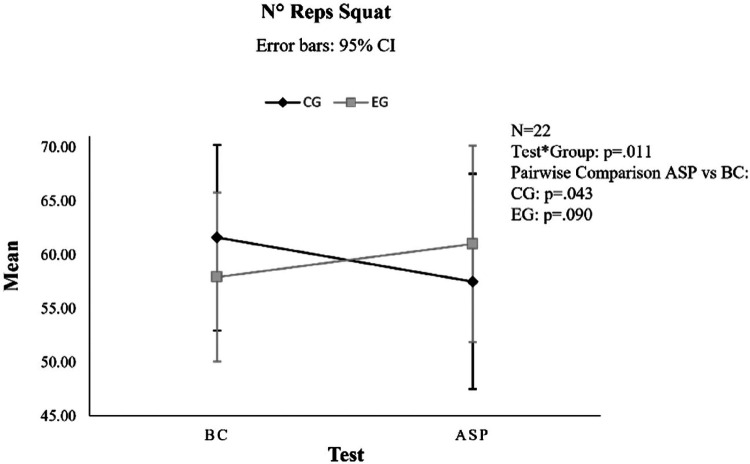
Interaction effect of the squat number of repetitions (N° reps). CG, control group; EG, experimental group; BC, baseline condition; ASP, after stretching protocol; CI, confidence interval.

No significant pre-post differences were observed for this outcome between BC and ASP but only in the interaction effect (main effect: *F*_1,20_ = .157, *p* = .696; interaction Test*Groups: *F*_1,20 _= 7.820, *p* = .011, *η*^2^ = .281). No differences were highlighted in the pairwise comparison between the groups (*F*_1,20_ = .289, *p* = .596). No differences were observed in the EG between BC and ASP (*F*_1,20 _= 3.170, *p* = .090). There was a significant worsening of CG between BC and ASP (*F*_1,20 _= 4.671, *p* = .043, *η*^2^ = .189; Figure 2).

### HRV-HRR

**Figure 3:**
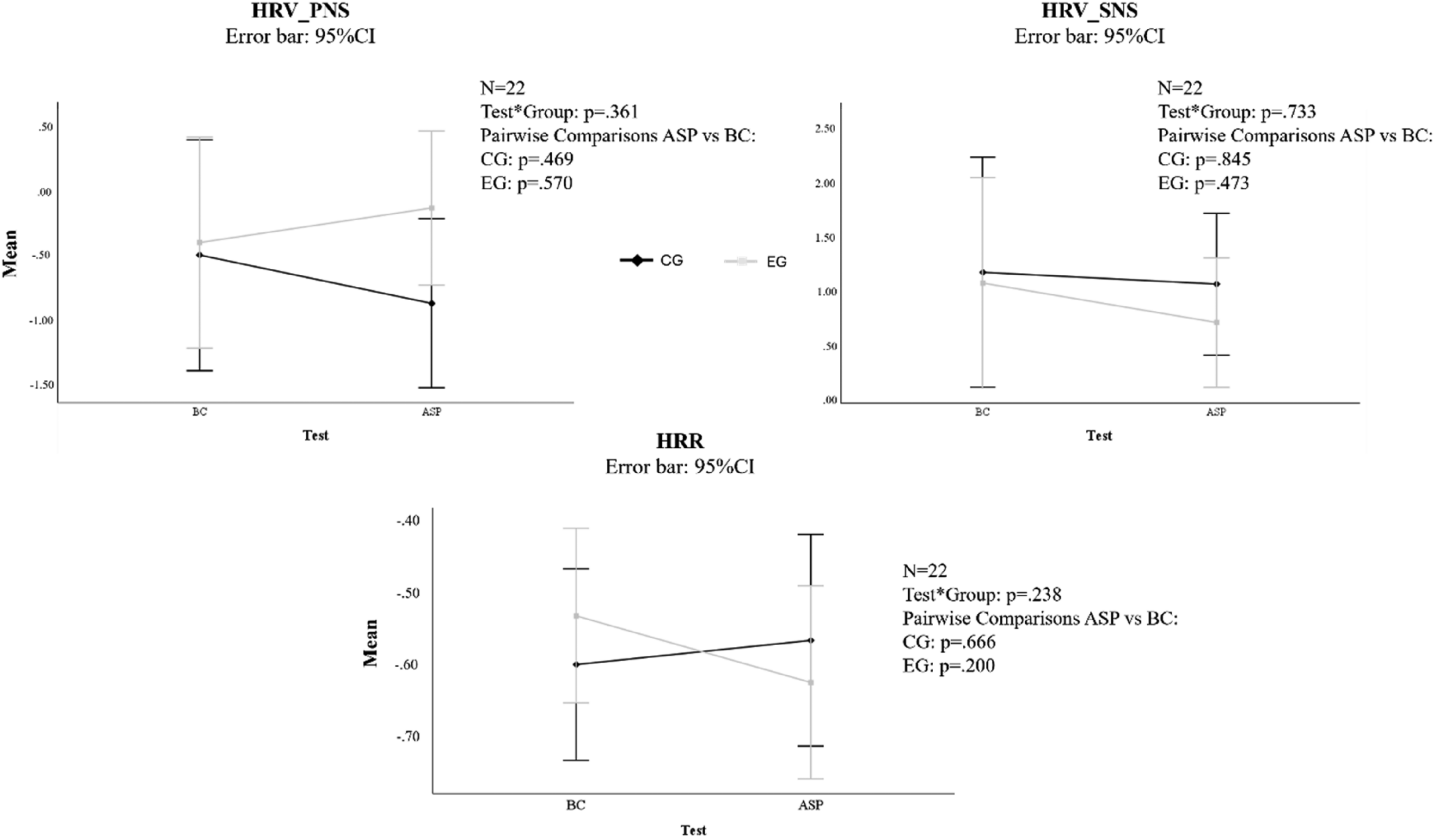
Trend in HRV-HRR for both groups. CG, control group; EG, experimental group; BC, baseline condition; ASP, after stretching protocol; CI, confidence interval; HRV, heart rate variability; HRR, heart rate recovery; PNS, parasympathetic nervous system; SNS, sympathetic nervous system.

No significant pre-post differences for this outcome were observed between BC and ASP, neither in parasympathetic nervous system (main effect: *F*_1,20_ = .024, *p* = .878; interaction Test*Groups: *F*_1,20_ = .874, *p* = .361), nor in sympathetic nervous system (main effect: *F*_1,20_ = .409, *p* = .530; interaction Test*Groups: *F*_1,20_ = .120, *p* = .733). No difference was revealed in the pairwise comparison between groups both parasympathetic nervous system (*F*_1,20 _= 1.243, *p* = .278) and sympathetic nervous system (*F*_1,20_ = .697, *p* = .41; Figure 3). Moreover, no significant pre-post differences for this outcome were observed between BC and ASP (main effect: *F*_1,20_ = .324, *p* = .576; Interaction Test*Groups: *F*_1,20 _= 1.479, *p* = .238), as well as between groups (*F*_1,20_ = .004, *p* = .952; Figure 3).

### Blood lactate concentration

**Figure 4:**
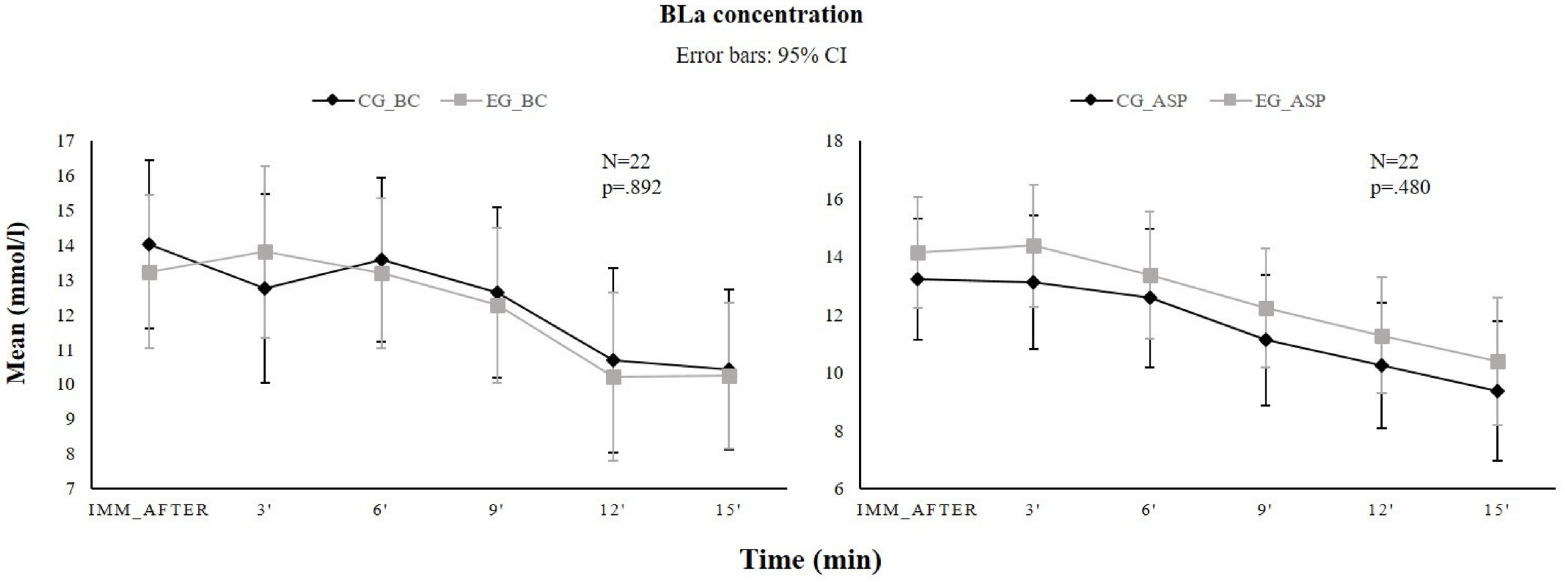
Time course (mean ± IC 95%) of blood lactate concentration before (left panel) and after (right panel) the stretching intervention for the control group (black line) and experimental group (gray line) at the following time points: immediately after (IMM_after), 3, 6, 9, 12, 15 min after the final exercise. BC, before stretching; ASP, after the stretching program; CI, confidence interval; BLa, blood lactate.

No significant pre-post differences in either group (main effect: *F*_1,20_ = .111, *p* = .743; interaction Test*Groups: *F*_1,20 _= 2.245, *p* = .150). Significant differences were observed in both groups in blood lactate clearance at the 12th minute compared to the first three readings (immediately after, 3rd and 6th minute) and at the 15th minute compared to the first four readings (immediately after, 3rd, 6th and 9th minute; main effect: *F*_1,20 _= 34.84, *p* < .001; interaction Time*Groups: *F*_1,20_ = .544, *p* = .742), but no difference between groups was revealed in subsequent measurements (*F*_1,20_ = .517, *p* = .480; Figure 4).

## Discussion

This exploratory study was designed to test whether and how a 4-week stretching program affects performance (i.e., number of bench press and squat repetitions) and bio-physiological markers. Our findings show that the stretching intervention was able to prevent the phenomenon of non-functional overreaching (6) on muscle performance. Given that the partial *η*^2^ indicated a significant decline in CG performance, it is possible to hypothesize that the observed decrease could be attributed to non-functional overreaching, as it can induce short-term performance decrements in the absence of adequate recovery during resistance training sessions. On the other hand, no worsening was observed in the EG, even if the neuroendocrine, neuromuscular, and biochemical mechanisms that determine non-functional overreaching are not clarified. This seems in contrast with literature where it has demonstrated a decrease in strength after stretching exercises (44–46). It is important to note that these researchers examined the effects of stretching on strength performance immediately following a stretching session. The absence of adequate muscle recovery time after stretching may have influenced strength performance, potentially due to morphological and neural responses (47). Therefore, it can be assumed that the stretching programs here affected maintaining muscle performance despite the same training load performed by the two groups. Given that stretching can maintain high levels of hypoxia-inducible factor 1-alpha, vascular endothelial growth factor A, and endothelial nitric oxide synthase in stretched limbs compared to non-stretched limbs (19, 48), the program we administered has likely contributed to maintaining high levels of vascularization by reducing muscle fatigue in the EG. Even though this statement needs to be confirmed with further investigations, we posit that the lack of statistical significance in the experimental group (*p* = 0.090) may stem from both the restricted number of exercises performed by participants and the abbreviated duration of the stretching session. This inference is supported by the literature, due that stretching exhibits a dose-dependent effect (49). The same behaviour is not observed in the bench press variable number of repetitions, probably due to a greater cardiovascular effort when larger muscle groups are stretched compared to smaller ones (50). This phenomenon seems to be due to a greater intramuscular pressure and peripheral resistance (51) which could stimulate angiogenesis and, consequently, contribute to an increase in hyperaemia in stretched muscles through endothelial mechano-transduction of shear force (19). The hypothesis of faster clearance of lactate in the group subjected to the stretching protocol was not verified and this fact could be explained by the bioavailable means of nitric oxide. The positive effects of physical activity on endothelial functions have been widely demonstrated: stress phenomena contribute to increasing the generation of nitric oxide and subsequently vasodilation and angiogenesis (52). Moreover, enhanced bioavailability of nitric oxide appears to be associated with specific attributes of the administered training session and distinct subgroups within the population. These include acute physical activities such as high-intensity interval training or aerobic exercises, sedentary individuals, healthy yet untrained persons, as well as participants afflicted with cardiovascular diseases or obesity (53). On the contrary, no significant changes in the production of nitric oxide were observed in athletes, probably due to a greater endothelium-dependent dilator reserve, unlike in untrained participants (54). The participants recruited in our study were resistance-trained men and women who had been following an intense training program for many years. Therefore, we hypothesize that the training carried out by our sample was not able to create an adaptation of the endothelium such as to constitute an enhanced endothelium-dependent dilation reserve. This aspect could be the cause that did not allow us to verify the hypothesis of faster lactate clearance in the EG, attributable to the buffering influence of nitric oxide. Finally, no significant changes in the HRR and HRV variables were observed between the groups and between the pre- and post-stretching programs. These results are in contrast to previous evidence in which beneficial effects of stretching in HRV were found (21, 22). These differences can be attributed to the distinct nature of the recruited sample in comparison to ours: individuals recruited were practitioners of strength training or bodybuilding, characterized by markedly restricted muscular flexibility and a lack of regular stretching practice for a minimum of two years. Instead, our sample declared that they stretched at least once a week, and it is likely that these regular stretching sessions could have led to an adaptation of the (micro)cardiovascular system, thus preventing changes in HRV or HRR from being highlighted. Therefore, the study we conducted prompts reflection on why stretching—in this particular category of participants and with these workloads—can improve performance while not affecting the bio-physiological mechanisms we have investigated. This opens up a path for further research toward a greater understanding of these separate effects, discovering the biomolecular basis underlying the efficacy of stretching in this particular effort and population.

## Practical applications

The results of this study highlight an effect of stretching on muscle fatigue, showing a deterioration of performance in the CG compared to the EG over a period of 4 weeks. Although this result requires further studies and in-depth analysis, it opens up interesting scenarios on the importance of stretching as an integral part of athletes' training programs and not just as a warm-up and cool-down activity.

## Limitations of the study

Although the study highlighted that stretching routine may mitigate muscle fatigue, it is possible to point out some limitations. Firstly, the calculated sample size does not seem adequate to observe an effect on the variable of interest “Number of squat repetitions” in the experimental group, given that the *p*-value observed was 0.09. Additionally, this aspect might be attributed to an insufficient overall workload in the stretching protocol used in this research that, in turn, would prevent us from observing effects on the bio-physiological variables considered. Furthermore, an involvement of the psychological sphere cannot be excluded. Actually, there are few studies focusing on the correlation between stretching and psychological variables [e.g., (55, 56)] while similar approaches such as hatha yoga (57–59) have shown to significantly improve psychological status. The study of psychological variables could point towards an understanding of why one group might have performed better than another in the absence of variation in biological parameters investigated. For what has been said so far and in order to fully understand the effects observed, future studies should take into account the psychological sphere, as well as a larger sample size and an increased duration and frequency of stretching sessions. This would provide valuable insights into the benefits of incorporating a stretching program into athletes' training periodization.

## Data Availability

The raw data supporting the conclusions of this article will be made available by the authors, without undue reservation.
